# Intraductal infection with H5N1 clade 2.3.4.4b influenza virus

**DOI:** 10.1128/jvi.01927-24

**Published:** 2025-01-31

**Authors:** Ericka Kirkpatrick Roubidoux, Victoria Meliopoulos, Brandi Livingston, Pamela H. Brigleb, Stacey Schultz-Cherry

**Affiliations:** 1 Department of Host Microbe Interactions, St. Jude Children's Research Hospital 5417 , Memphis, Tennessee, USA; The University of Arizona, Tucson, Arizona, USA

**Keywords:** influenza, emerging, intraductal

## LETTER

In March 2024, highly pathogenic avian influenza (HPAI) H5N1 of the clade 2.3.4.4b was detected in dairy cows in Texas and has since been detected in several other U.S. states (1). Virus has been detected within cow’s milk, indicating that the mammary epithelium may support viral replication (2). Virus has also been detected on milking machines, leading to a hypothesis that influenza is spreading through fomites from udder to udder instead of the intranasal route (3, 4). There have been studies using cows to better understand mammary infections; however, the cow model is costly and limited (1, 5). We sought to establish a model for intramammary infections of H5N1 and H1N1 influenza virus in mice.

To test the hypothesis that intraductal inoculation is a route of infection, lactating C57Bl/6 mice were inoculated with 0.5× mean 50% lethal dose (mLD_50_) of A/bovine/Ohio.B24OSU-439/2024 (H5N1) influenza virus into actively lactating nipples (an average of seven lactating nipples per mouse) using a 33G needle. The dams and respective pups were monitored daily to assess clinical signs, weight loss, and survival.

**Fig 1:**
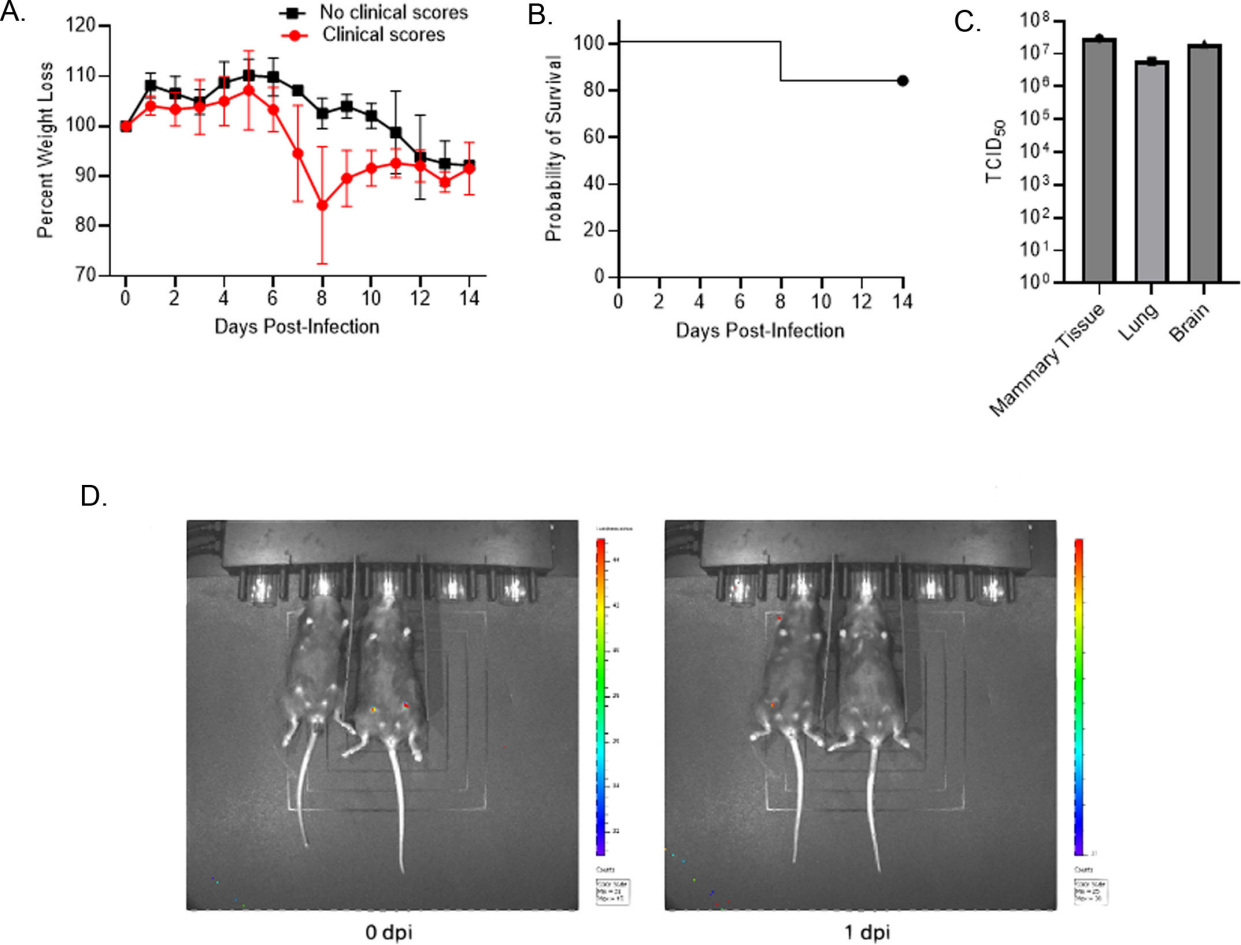
H5N1 and H1N1 intraductal infections. (**A**) Weight loss of mice intraductally infected with H5N1. (**B**) Survival of mice intraductally infected with H5N1. (**C**) Viral titers in various tissues of the mouse that succumbed to H5N1 infection. (**D**) H1N1 intraductal infections. The mouse on the left was uninfected, while the mouse on the right received H1N1 via intraductal injection. The image on the left is at 0 dpi, while the image on the right is at 1 dpi.

After intraductal infection, three out of six dams exhibited clinical scores and trended toward increased weight loss ranging from 5% to 30% with one mouse succumbing to infection (Fig. 1A and B). The deceased mouse had high viral titers in mammary glands, lungs, and brain, suggesting systemic spread (Fig. 1C). Weight loss in the dams may have been associated with loss of appetite from the infection, we also hypothesize that the lactating mice suffered from “milk drop syndrome,” where they failed to produce milk. Additionally, the mice that demonstrated the most significant weight loss lost their pups (*n* = 11/16), which did not test positive for virus. Surviving pups (*n* = 5) in this model did not test positive for virus at 1 or 3 days post-infection (dpi), indicating that the model may not be ideal for evaluating transmission of the virus in milk from dams to pups. All pups from dams without weight loss survived (14 out of 14).

To test if mammary tropism is virus subtype specific, we also performed intraductal inoculations in mice using the pandemic H1N1 virus A/California/04/2009. While the injections were successful, the virus was no longer detectable at 1 dpi (Fig. 1D). These results suggest that H5N1 viruses are more equipped to replicate in mammary glands.

Intraductal inoculations in the mouse model can be used to evaluate lethality and viral spread of H5N1, but not H1N1, viruses. This information is important to our understanding of tissue tropism and can be used to assess if antiviral treatments prevent infections in the mammary gland.
